# Predictive models for dysphagia in older adults: a systematic review and meta-analysis

**DOI:** 10.3389/fpubh.2026.1792380

**Published:** 2026-04-02

**Authors:** Shujun Fan, Bingbing Chen, Chuan Yin, Yu Hao, Wanning Liu, Yu Zhang, Qin Liu, Zhe Zhang

**Affiliations:** 1 Nursing Department, Fuyang Normal University Second Affiliated Hospital, Fuyang, Anhui, China; 2 Fuyang Medical School, Fuyang Normal University, Fuyang, Anhui, China; 3 Department of Oncology, The Second Affiliated Hospital of Anhui Medical University, Hefei, Anhui, China

**Keywords:** dysphagia, meta-analysis, nursing, predictive models, systematic review

## Abstract

**Introduction:**

Dysphagia is a common condition among older adults, closely linked to aging and neurodegenerative diseases. It can lead to malnutrition, frailty, and aspiration pneumonia, thereby impairing quality of life and clinical outcomes. Although various interventions may improve swallowing function, early identification of high-risk individuals remains challenging. Existing predictive models show inconsistent performance and lack systematic evaluation. This study aimed to systematically review and assess predictive models for dysphagia risk in older adults.

**Methods:**

A comprehensive search was conducted across CNKI, the Chinese Science and Technology Journal Database, the Chinese Biomedical Literature Database, Wanfang Data, PubMed, Web of Science, and the Cochrane Library, covering studies published up to September 15, 2025.

**Results:**

Of 7,113 records identified, 17 met inclusion criteria, with only two performing external validation. Reported AUCs ranged from 0.682 to 0.926, and all studies showed a high overall risk of bias. The pooled AUC from the meta-analysis was 0.82 (95% CI: 0.77–0.88). Common predictors included advanced age, smoking history, reduced self-care ability, polypharmacy, frailty, malnutrition, cognitive impairment, and poor oral health.

**Discussion:**

Overall, predictive modeling for dysphagia in older adults remains in an early stage, limited by methodological flaws and insufficient external validation. Future research should follow PROBAST standards and conduct large, multicenter validations to improve model reliability and clinical utility.

## 1 Introduction

Swallowing refers to the sequential transport of food or liquid from the oral cavity through the pharynx to the stomach (1). Its normal function depends on the precise coordination of skeletal and smooth muscles, the integrity of soft tissues, and the orderly control of the nervous system. Any abnormalities in anatomical structure, neural function, or physiological mechanisms may disrupt this process and lead to dysphagia (2). Epidemiological studies estimate that the prevalence of swallowing difficulty among older adults ranges from 11.8 to 32.2% (2, 3). This high prevalence is partly due to the natural aging process, including muscle atrophy, reduced tissue elasticity, and diminished sensory reflexes (4). Furthermore, dysphagia frequently manifests as a concomitant indicator within the spectrum of neurodegenerative disorders and chronic non-communicable diseases, such as stroke, Parkinson's disease, and head and neck pathologies (5). Given that age-related swallowing decline and its associated adverse outcomes are predominantly concentrated in the oropharyngeal phase, the present study specifically focuses on oropharyngeal dysphagia in the older adult population.

The adverse outcomes of dysphagia deserve close attention. Studies have shown that older adults with dysphagia have about a 4.8-fold higher risk of malnutrition compared with those without the condition (6). Oropharyngeal dysphagia directly reduces protein and energy intake, which may cause negative nitrogen balance and accelerate skeletal muscle protein breakdown, contributing to sarcopenia and frailty (7, 8). Disruption of the nutrition–muscle axis further weakens gait stability and physiological reserve, increasing the risk of falls, disability, and dependence. In addition, incomplete epiglottic inversion, reduced laryngeal elevation, and delayed pharyngeal response can compromise airway protection, resulting in frequent aspiration and a higher incidence of aspiration pneumonia, which in turn increases hospital readmission and short-term mortality (2, 9, 10).

Fortunately, dysphagia can be prevented and improved through several nonpharmacological measures, including swallowing training, electrical stimulation therapy, health education, and multimodal interventions (11). The key lies in the early identification of older adults at high risk of dysphagia (2). However, traditional screening tools mainly detect existing functional impairment (2). For instance, the water-swallow test (WST) is widely used for its simplicity but is only sensitive to liquid aspiration and cannot detect silent aspiration (2, 12). The EAT-10 questionnaire shows good reliability and validity but applies only to conscious individuals with eating experience (2). The videofluoroscopic swallow study (VFSS), regarded as the diagnostic gold standard, is limited by radiation exposure and cannot be used repeatedly (2).

In recent years, studies have begun to develop prospective risk prediction models for dysphagia, covering populations such as community dwellers, hospitalized patients, and those with stroke (2, 13, 14). Predictive variables have expanded from demographic characteristics to include comorbidities, medication use, oral health, self-care ability, educational level, and biomarkers such as albumin and neutrophil count (2, 13–15). These models aim to estimate individualized risk and support stratified prevention. However, differences in model discrimination, calibration, bias control, and external validation remain substantial, and reporting standards vary across studies, which limits their clinical application. Systematic reviews on predictive factors for dysphagia in older adults are still lacking. Therefore, evidence-based synthesis of existing prediction tools is urgently needed to summarize current model development, evaluate predictive performance and clinical feasibility, and provide a foundation for future model optimization and prospective validation.

## 2 Materials

### 2.1 Design

This systematic review was conducted in strict accordance with the Transparent Reporting of Multivariable Prediction Models for Individual Prognosis or Diagnosis: Checklist for Systematic Reviews and Meta-Analyses (TRIPOD-SRMA) guidelines (16). The study protocol was prospectively registered on the International Prospective Register of Systematic Reviews (PROSPERO; registration number CRD420251165034).

### 2.2 Search strategy

A comprehensive literature search was conducted across seven Chinese and English databases, including CNKI, VIP, SinoMed, Wanfang Data, PubMed, Web of Science, and the Cochrane Library, to identify relevant studies published from database inception to September 15, 2025. The search strategy combined controlled vocabulary terms (e.g., MeSH terms) and free-text keywords related to dysphagia, older adults, and predictive models. The detailed search strategies for each database are provided in Supplementary Table 1. In addition to peer-reviewed journal articles, databases that index academic dissertations and theses (e.g., CNKI and Wanfang Data) were also searched, allowing the identification of relevant gray literature. Studies retrieved from these sources were considered eligible if they met the predefined inclusion criteria. Furthermore, the reference lists of all included studies were manually screened to identify additional potentially eligible studies that may not have been captured through the database searches.

### 2.3 Inclusion and exclusion criteria

Inclusion criteria: (1) Study design: prospective or retrospective cohort studies, case–control studies, or cross-sectional studies; (2) Population: older adults aged 60 years or above; (3) Predictive models: studies that developed dysphagia risk prediction models using multivariable statistical methods (e.g., logistic regression, Cox regression, or machine learning) and reported at least one performance metric, such as AUC, sensitivity, specificity, calibration, or discrimination. Exclusion criteria: (1) Publication type: reviews, conference abstracts, or study protocols; (2) Studies including participants under 60 years without separate analysis for those aged 60 or above; (3) Single-predictor studies: studies examining only one variable without developing a multivariable predictive model; (4) Full text unavailable despite exhaustive retrieval attempts; (5) Incomplete data: missing essential modeling information (e.g., regression coefficients, variable definitions, or performance metrics) with unsuccessful attempts to contact the authors; (6) Diagnostic accuracy studies: studies aimed solely at validating new screening or diagnostic tools for dysphagia rather than developing predictive models.

### 2.4 Study screening and data extraction

Literature screening was independently performed by two researchers. Duplicates were first removed, followed by an initial screening based on titles and abstracts, and a subsequent full-text review. Reference lists of included studies were manually traced to identify any additional eligible studies. Discrepancies at any stage were resolved through discussion and consensus. For data extraction, a predesigned form based on the CHARMS checklist (17) was used. The first researcher systematically recorded details including author, year, country, disease spectrum, age, study design, number of events/total sample in the training set, outcome definition, diagnostic criteria, modeling strategy, variable selection methods, number of models, and reported performance metrics. The second researcher independently verified all entries to ensure accuracy, with any disagreements resolved through consultation.

### 2.5 Quality assessment

The methodological quality and applicability of included predictive models were assessed using the Prediction model Risk Of Bias Assessment Tool (PROBAST) (18). PROBAST comprises two core domains: risk of bias and applicability. The risk of bias domain includes participants, predictors, outcome, and analysis, while the applicability domain focuses on participants, predictors, and outcome. A domain was rated as low risk only if all signaling questions were answered “yes” or “probably yes.” If any question was “no information” and the remainder were “yes” or “probably yes,” the domain was rated as unclear. Any “no” or “probably no” response, regardless of other answers, resulted in a high-risk rating. Overall risk of bias was conservatively integrated: only if all domains were low risk was the overall rating low; any high-risk domain led to an overall high-risk classification; if unclear domains existed without any high-risk domain, the overall rating was unclear. Applicability was similarly assessed per domain as low concern, high concern, or unclear, with overall applicability determined by the highest concern across domains. Two researchers performed assessments independently, cross-checked results, and submitted unresolved disagreements to a third-party adjudicator to ensure objectivity and reproducibility.

### 2.6 Data synthesis

Statistical analyses were performed using Stata version 17.0 (StataCorp LLC, College Station, TX, United States). Predictors that were reported in at least three studies were considered eligible for quantitative synthesis. For these predictors, pooled effect estimates were calculated as odds ratios (ORs) with corresponding 95% confidence intervals (95% CIs). Given the anticipated clinical and methodological heterogeneity among the included studies, including differences in study populations (e.g., older adults with different underlying diseases), study designs, and definitions of predictors, a random-effects model was applied to pool the effect estimates. Statistical heterogeneity was assessed using Cochran's *Q*-test and the *I*^2^ statistic, with *I*^2^ values of 25, 50, and 75% indicating low, moderate, and high heterogeneity, respectively. All statistical tests were two-sided, and a *p* value < 0.05 was considered statistically significant.

## 3 Results

A total of 7,113 records were identified, of which 5,319 remained after duplicate removal using EndNote. Screening of titles and abstracts led to the exclusion of 5,180 records, leaving 139 articles for full-text assessment. Ultimately, 17 studies met the inclusion criteria (2, 3, 10, 13–15, 19–29). These studies were published between 2021 and 2025. Study populations were drawn from China (*n* = 15), Spain (*n* = 1), and Italy (*n* = 1).

**Figure 1:**
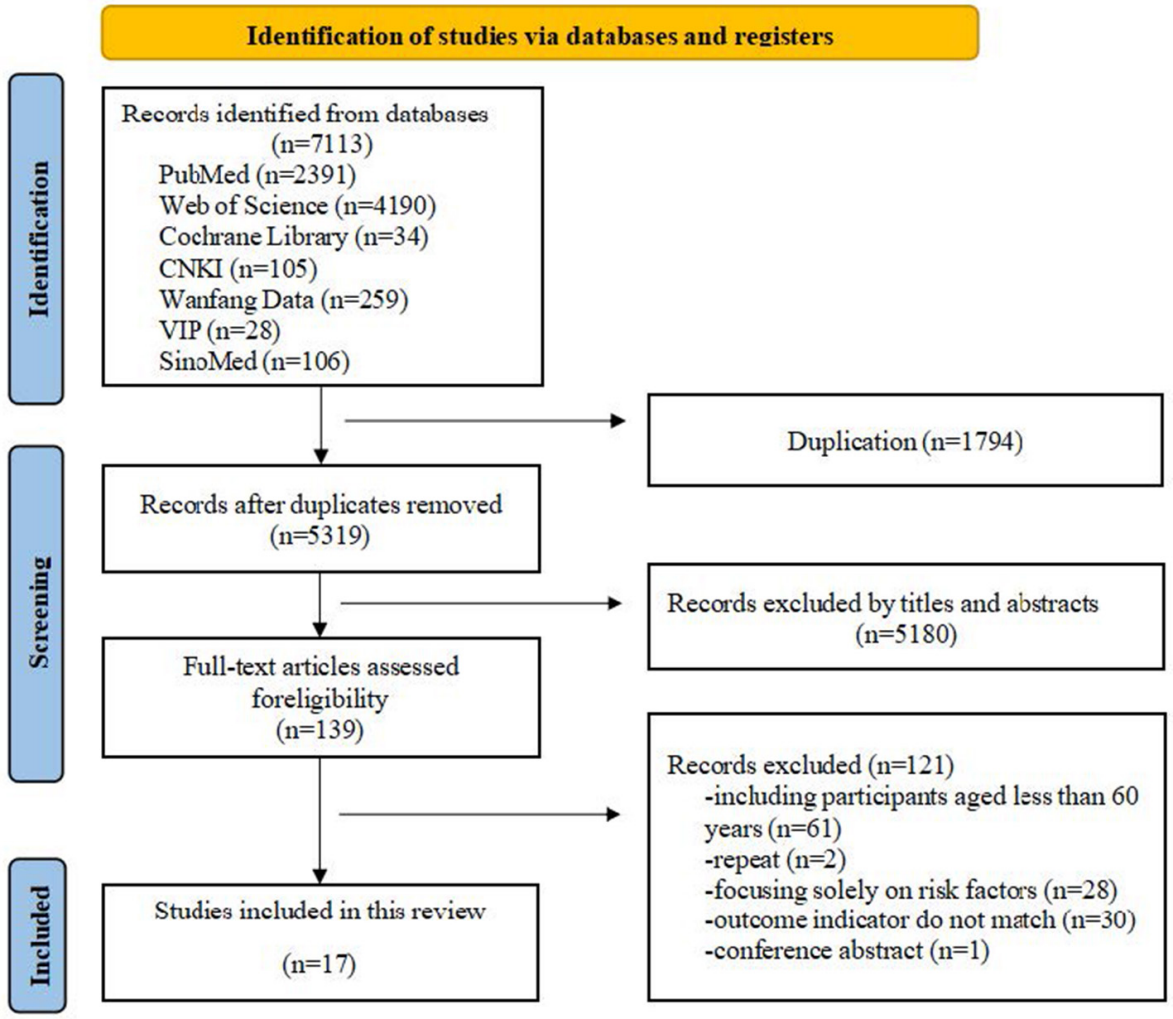
Flow diagram of study selection.

Regarding predictive settings, 13 studies were conducted in hospital environments (3, 13–15, 19–24, 26, 28, 29), three in long-term care facilities (10, 25, 27), and one in a community-dwelling population (2). Within the hospital subgroup, most studies further focused on specific patient populations, including hypopharyngeal cancer (19), mechanically ventilated ICU patients (20), individuals with cognitive impairment (21), stroke (14, 15), and chronic obstructive pulmonary disease (22, 26). In terms of dysphagia assessment tools, the Kubota Water Swallowing Test was the most frequently employed (*n* = 10) (2, 3, 13, 15, 19, 21, 22, 24, 26, 27). Other instruments included the EAT-10 questionnaire (*n* = 3) (2, 23, 25), the Standardized Swallowing Assessment (*n* = 3) (10, 20, 29), the Volume-Viscosity Swallow Test (*n* = 1) (28), and the Functional Oral Intake Scale (*n* = 1) (14). The study selection process is illustrated in Figure 1, and baseline characteristics of the included studies are summarized in Table 1.

**Table 1:** Overview of basic data of the included studies (*n* = 17).

References	Country	Disease	Age	Study design	Sample size (training set)		Diagnostic criteria
					Cases size	Total sample size	
Ge (19)	China	Hypopharyngeal cancer	60–84	Retrospective cohort	41	70	WST
Cao and Wang (20)	China	ICU mechanical ventilation	≥60	Cross-sectional	18	180	SSA
Xie and Chen (21)	China	Cognitive impairment	≥60	Retrospective cohort	42	150	WST
Liu et al. (15)	China	Stroke	60–96	Retrospective cohort	—	1,823	WST
Gan (22)	China	COPD	≥60	Retrospective cohort	—	134	WST
Yu et al. (23)	China	Hospitalized older adults	65–93	Prospective cohort study	70	343	EAT-10
Chen (24)	China	Hospitalized older adults	≥65	Retrospective cohort	—	1,772	WST
Yan et al. (25)	China	Retired older adults	65–110	Cross-sectional	106	251	EAT-10
Peng et al. (3)	China	Hospitalized older adults	≥65	Retrospective case-control study	1,009	3,134	WST
Chen et al. (26)	China	COPD	>60	Retrospective cohort	120	250	WST
Chen et al. (27)	China	Care institutions for older adults	≥60	Cross-sectional	182	558	WST
Martin-Martinez et al. (28)	Spain	Older adults with acute diseases	—	Retrospective cohort	—	2,326	V-VST
Wang et al. (29)	China	Tracheal intubation under general anesthesia	≥65	Cross-sectional	46	327	SSA
Lin et al. (13)	China	Frail older inpatients	≥65	Cross-sectional	103	287	WST
Mattavelli et al. (14)	Italy	Ischaemic stroke patients	75.8 ± 12.4	Retrospective cohort	82	226	FOIS
Qiu et al. (2)	China	Community-dwelling older adults	≥65	Cross-sectional	320	2,561	WST; EAT-10
Liu et al. (10)	China	Care facility residents	≥60	Cross-sectional	281	409	SSA

WST, Kubota water swallowing test; SSA, standardized swallowing assessment; V-VST, volume-viscosity swallow test; FOIS, functional oral intake scale.

### 3.1 Basic characteristics of risk prediction models

Table 2 provides a systematic overview of the key characteristics of the included studies. Six studies (15, 21, 22, 24, 26, 28) reported multiple predictive models, resulting in a total of 30 dysphagia risk prediction tools. Fourteen studies conducted internal validation only (2, 3, 13, 14, 19, 20, 22–29), while two studies performed both internal and external validation (10, 15). Most studies employed univariable analysis for variable selection and constructed models using binary or multivariable logistic regression (2, 13–15, 19–22, 24–29). In the study by Yu et al. (23), a Cox regression model was used to develop the final risk prediction model. Additionally, machine learning techniques—including XGBoost, weighted k-nearest neighbors, support vector machines, random forests, and BP neural networks—were applied in studies by Chen (24), Peng et al. (3), and Martin-Martinez et al. (28). Six studies assessed model calibration using the Hosmer-Lemeshow test (2, 10, 13, 20, 27, 29), and nine studies reported at least one measure of sensitivity, specificity, or overall accuracy (2, 3, 14, 20, 22, 24, 27–29). Reported AUC or C-statistics ranged from 0.682 to 0.926.

**Table 2:** Model establishment status.

Authors	Model development			Model performance			Validation method	Predictors
	Variable selection	Model development method	Model number	AUC/C-index (95% CI)	Calibration method	Specificity/ sensitivity/ accuracy		
Ge (19)	(1) Univariate analysis	(1) Multivariate logistic regression analysis	1	B: 0.899 (0.863–0.935)	Graphical calibration (calibration plot)	—	a	Age; smoking history; surgical method; tumor T staging; postoperative radiotherapy
Cao and Wang (20)	(1) Univariate analysis	(1) Multivariate logistic regression analysis	1	A: 0.736 (0.674–0.791)	Hosmer-Lemeshow test	0.79/0.60/84.44%	a	Age; APACHE II score; number of intubation attempts; mechanical ventilation time; airbag compression; use neuromuscular blockers; history of head and neck radiotherapy
Xie and Chen (21)	(1) Univariate analysis	(1) Multivariate logistic regression analysis	2	A: 0.813 (0.753–0.873) 0.788 (0.748–0.828)	—	—	—	Daily life activity ability; swallowing training; number of medication types; age; weakness
Liu et al. (15)	(1) Univariate analysis	(1) Multivariate logistic regression analysis	4	B: 0.902; 0.883; 0.877; 0.868	Graphical calibration (calibration plot)	—	a + b	Age; drink wine; hypertension; diabetes; muscle strength level; stroke side; brain injury area; neutrophils; serum albumin; serum potassium
Gan (22)	(1) Univariate analysis	(1) Multivariate logistic regression analysis	2	B: 0.926 (0.892–0.966) 0.904 (0.860–0.948)	—	90.3%/80.0%/89.4% 80.0%/88.9%/—	a	Difficulty in breathing; severity of illness; tooth loss; eating posture; partial pressure of carbon dioxide; type of medication used; smoking history; nutritional status; concentrate on eating
Yu et al. (23)	(1) Univariate analysis (2) LASSO regression screening	(1) Cox multivariate regression	1	B: 0.74 (0.69–0.79)	Graphical calibration (calibration plot)	—	a	Age; wearing dentures; daily life activity ability; cerebral vascular disease; coronary heart disease; tumor
Chen (24)	(1) Univariate analysis (2) LASSO regression screening	(1) Multivariate logistic regression analysis (2) K-nearest neighbor (3) Support vector machine (4) Random forest (5) Artificial neural networks (6) eXtreme gradient boosting	6	A: 0.8814 0.8741 0.8527 0.8815 0.8795 0.8599	Graphical calibration (calibration plot)	—/0.6369/0.8434 —/0.6012/0.8289 —/0.5655/0.8276 —/0.6438/0.8487 —/0.6250/0.8447 —/0.5476/0.8316	a	Age; BMI; self-evaluation of health status; weakness; daily living ability; oral scoring; community activities; assisting with household chores; cognitive function; primary caregiver
Yan et al. (25)	(1) Univariate analysis	(1) Multivariate logistic regression analysis	1	A: 0.796 (0.738–0.855)	Graphical calibration (calibration plot)	—	a	Long term use of sedatives and hypnotics; history of coughing while drinking water; daily life activity ability
Peng et al. (3)	(1) Spearman correlation screening (2) LASSO-logistic regression	(1) XGBoost	1	B: 0.855	—	0.881/0.613/0.795	a	Muscle atrophy; gender; age; caregivers; weakness; oral scoring; daily life activity ability; frequency of community activities; depression; cognitive function
Chen et al. (26)	(1) Univariate analysis	(1) Multivariate logistic regression analysis	2	A: 0.682 (0.620–0.739) 0.747 (0.689–0.800)	—	—	a	Tooth loss situation; nutritional status; cognitive impairment; oral weakness; severity of COPD
Chen et al. (27)	(1) Univariate analysis	(1) Binary logistic regression	1	A: 0.912 (0.886–0.938)	Hosmer-Lemeshow test	0.851/0.830/—	a	Age; smoking history; oral denture condition; assistance with dining in care institutions for older adults; daily eating preference tends to be hot; functional oral intake level; oral health-related self-efficacy; oral health score
Martin-Martinez et al. (28)	(1) Linear approach: univariate/multivariate analysis (2) Non-linear approach: XGBoost	(1) Linear approach: multivariable logistic regression (2) Non-linear approach: XGBoost gradient boosting	2	B: 0.840 (0.829–0.867) 0.734 (0.713–0.755)	—	0.416/0.940/— 0.191/0.964/—	a	Demographic data; diagnostic code (ICD-10); functional and frailty scale/index; administrative data; related drugs for swallowing disorders
Wang et al. (29)	(1) Univariate analysis	(1) Binary logistic regression	1	A: 0.833 (0.768–0.898) B: 0.822 (0.737–0.907)	Hosmer-Lemeshow test	81.5%/76.1%/—	a	Age; retention time of gastric tube; difficulty level of tracheal intubation; nebulization after extubation; anesthesia risk level; weakness
Lin et al. (13)	(1) Univariate analysis	(1) Multivariate logistic regression analysis	1	A: 0.875 (0.833–0.916)	Hosmer-Lemeshow test	—	a	Age; multiple use of medication; history of suffocation; nutrition; oral health self-efficacy; oral health status
Mattavelli et al. (14)	(1) Univariate analysis	(1) Multivariate logistic regression analysis	1	A: 0.880 (0.83–0.92)	—	87.5%/73.2%/—	a	Severity of stroke; location of lesion (frontal lobe insula); infarct size; pre stroke mRS; therapeutic intervention; education level
Qiu et al. (2)	(1) Univariate analysis	(1) Multivariate logistic regression analysis	1	A: 0.709 (0.679–0.739) B: 0.693 (0.640–0.747)	Hosmer-Lemeshow test	0.50/0.769/— 0.52/0.726/—	a	Maximum tongue pressure; number of molars; pneumonia; daily life activity ability; sarcopenia; age; neurological disorders; rheumatic diseases
Liu et al. (10)	(1) LASSO regression	(1) Multivariate logistic regression analysis	1	A: 0.800 (0.755–0.844) B: 0.824 (0.747–0.901)	Hosmer–Lemeshow test + calibration curve	—	a + b	Stroke; history of sputum aspiration; daily life activity ability; nutrition; texture improvement food

A, development cohort; B, validation cohort; a, internal validation; b, external validation.

### 3.2 Risk estimation

The Prediction model Risk Of Bias Assessment Tool (PROBAST) was employed to evaluate the risk of bias and applicability of the included studies (18). Table 3 summarizes the assessment results: all studies demonstrated good applicability but were uniformly rated as having a high overall risk of bias. Specific sources of bias can be categorized into four domains: (1) Participants: Risk predominantly arose from single-center studies with limited sample sizes, resulting in clear selection bias and failure to meet representativeness criteria. (2) Predictors: While all studies provided detailed information on the measurement tools and scoring standards for predictor variables, none reported whether assessors were blinded during measurement, nor could the temporal relationship between predictor and outcome measurement be inferred from the reports. Consequently, observer bias or reverse causation could not be excluded, and this domain was rated as high risk. (3) Outcomes: Standardized instruments such as the WST, SSA, or FOIS were commonly used to assess swallowing function with clear cut-offs. However, no study provided evidence regarding whether outcome assessors were blinded to predictor information or the temporal order of predictor and outcome assessment. This domain was therefore uniformly rated as unclear risk of bias. (4) Analysis: Seven studies had insufficient sample sizes and did not meet the events-per-variable (EPV) ≥20 criterion (13, 19–22, 25, 29). Fifteen studies handled continuous variables inappropriately (2, 3, 10, 13, 14, 19–27, 29). Thirteen studies did not report methods for handling missing data (2, 10, 13, 14, 19–23, 26–29), while one study acknowledged missing values and implemented supplementary measures (25). All studies treated participants as independent observations, failing to account for clustering within institutions (e.g., across different nursing homes or hospitals) or for competing events such as death, loss to follow-up, or transfers. As a result, all studies were classified as high risk of bias in the analysis domain.

**Table 3:** The risk of bias and applicability.

Authors	Risk of bias				Applicability			Overall	
	Participants	Predictors	Outcome	Analysis	Participants	Predictors	Outcome	Risk of bias	Applicability
Ge (19)	H	H	N	H	L	L	L	H	L
Cao and Wang (20)	H	H	N	H	L	L	L	H	L
Xie and Chen (21)	H	H	N	H	L	L	L	H	L
Liu et al. (15)	L	H	N	H	L	L	L	H	L
Gan (22)	H	H	N	H	L	L	L	H	L
Yu et al. (23)	L	H	N	H	L	L	L	H	L
Chen (24)	L	H	N	H	L	L	L	H	L
Yan et al. (25)	H	H	N	H	L	L	L	H	L
Peng et al. (3)	L	H	N	H	L	L	L	H	L
Chen et al. (26)	H	H	N	H	L	L	L	H	L
Chen et al. (27)	H	H	N	H	L	L	L	H	L
Martin-Martinez et al. (28)	H	H	N	H	L	L	L	H	L
Wang et al. (29)	H	H	N	H	L	L	L	H	L
Lin et al. (13)	H	H	N	H	L	L	L	H	L
Mattavelli et al. (14)	H	H	N	H	L	L	L	H	L
Qiu et al. (2)	H	H	N	H	L	L	L	H	L
Liu et al. (10)	H	H	N	H	L	L	L	H	L

L, low risk/high applicability; H, high risk/low applicability; N, unclear.

### 3.3 Results of statistical analysis

#### 3.3.1 Meta-analysis of validation models included in the review

**Figure 2:**
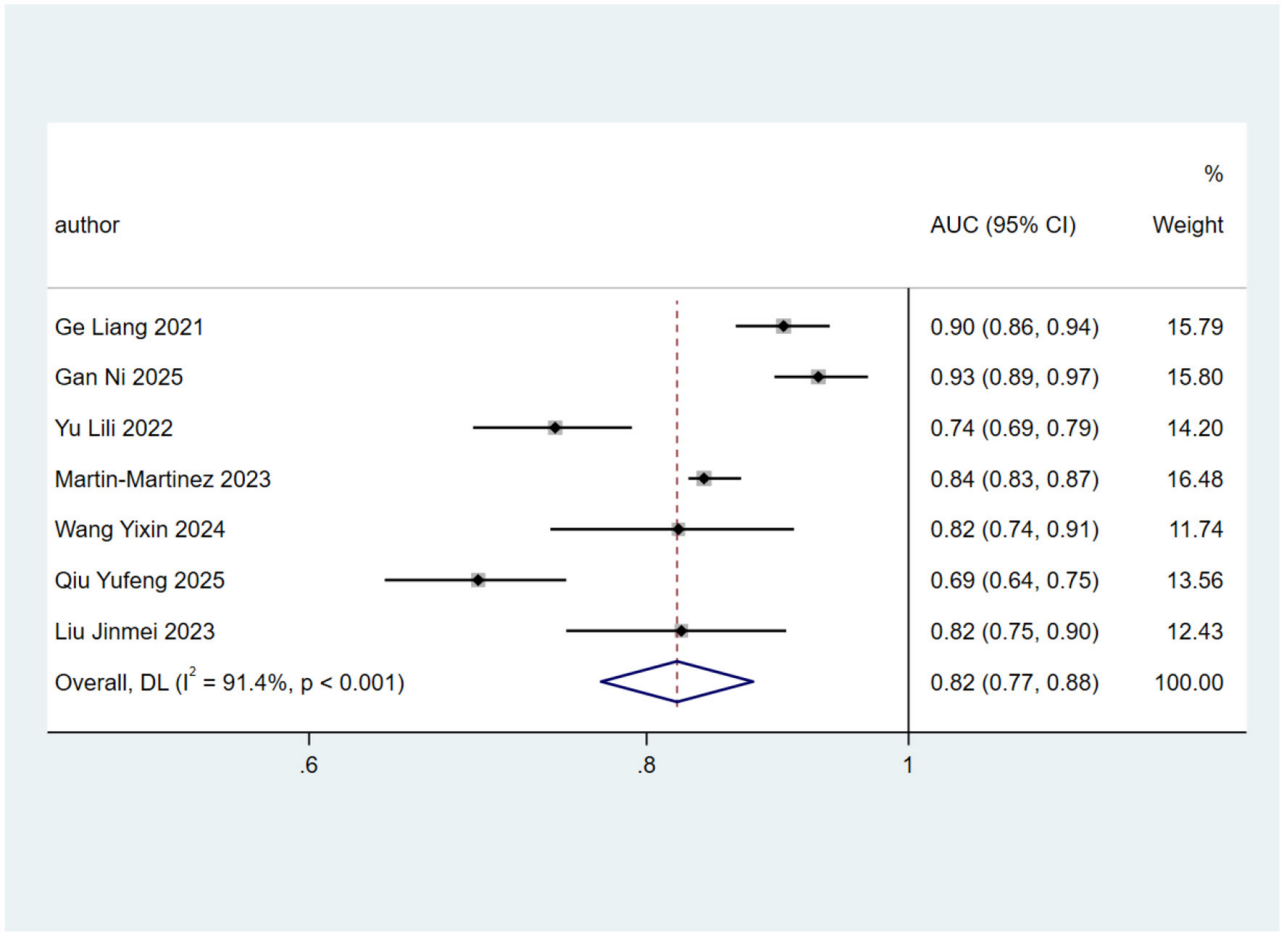
Forest plot of the random effects meta-analysis of pooled AUC estimates for seven validation models. Weights are from random-effects model.

Owing to the absence of key development details, such as confidence intervals for the AUC, in some studies (3, 15), only seven articles met the inclusion criteria (2, 10, 19, 22, 23, 28, 29). To avoid bias from duplicate use of the same datasets (22, 28), only the model with the best reported predictive performance from each study was included in the meta-analysis. Heterogeneity testing revealed *I*^2^ = 91.4% (*p* < 0.001), indicating substantial between-study variability. A random-effects model was therefore applied, yielding a pooled AUC of 0.82 (95% CI: 0.77–0.88; Figure 2).

#### 3.3.2 Meta-analysis of common predictors

Meta-analyses were conducted for predictors reported in three or more studies, using random-effects models (Table 4). The study by Martin-Martinez et al. (28) was excluded due to unavailable extractable statistics. (1) Age: Eleven studies (2, 3, 13, 15, 19–21, 23, 24, 27, 29) reported the association between age and dysphagia. The study by Xie and Chen (21) did not specify whether age was treated as a categorical or continuous variable and was therefore excluded. Meta-analysis was performed on nine studies (2, 3, 13, 19, 20, 23, 24, 27, 29) that reported age as a categorical variable. Advanced age was significantly associated with increased dysphagia risk (OR = 2.67, 95% CI = 2.12–3.38, *p* < 0.001). (2) Smoking: Three studies (19, 22, 27) examined smoking history as a predictor. Smoking was significantly associated with higher dysphagia risk (OR = 2.95, 95% CI = 1.94–4.49, *p* < 0.001). (3) Functional independence: Seven studies (2, 3, 10, 21, 23–25) assessed the impact of functional independence. Meta-analysis stratified by variable type (categorical/continuous) indicated that impaired self-care significantly increased the risk of dysphagia. (4) Polypharmacy: Three studies (13, 21, 22) reported polypharmacy as a predictor, showing it as a significant risk factor (OR = 4.37, 95% CI = 2.11–9.06, *p* < 0.001). (5) Frailty: Four studies (3, 21, 24, 29) identified frailty as a significant risk factor for dysphagia (OR = 4.28, 95% CI = 2.28–8.04, *p* < 0.001). (6) Malnutrition: Four studies (10, 13, 22, 26) evaluated malnutrition, but pooled results were not statistically significant (OR = 1.71, 95% CI = 0.76–3.84, *p* = 0.194). (7) Cognitive impairment: Three studies (3, 24, 26) demonstrated that cognitive impairment significantly increased dysphagia risk (OR = 1.54, 95% CI = 1.19–2.00, *p* = 0.001). (8) Oral health: Four studies (3, 13, 24, 27) assessed oral health status, showing that poor oral health was significantly associated with increased dysphagia risk (OR = 0.87, 95% CI = 0.84–0.91, *p* < 0.001). Forest plots for these analyses are provided in the Supplementary material.

**Table 4:** Meta analysis results of predictive factors.

Predictors	Number of studies	Heterogeneity test		Meta-analysis		
		*I*^2^ (%)	*p*-value	OR (95% CI)	*Z*	*p*-value
Age (2, 3, 13, 19, 20, 23, 24, 27, 29)	9	25.5	0.217	2.67 (2.12, 3.38)	8.29	< 0.001
Smoking history (19, 22, 27)	3	0.3	0.367	2.95 (1.94, 4.49)	5.08	< 0.001
**Self-care ability** **(****2**, **3**, **10**, **21**, **23****–****25****)**						
Categorical variable (3, 21, 23, 24)	4	36.6	0.193	4.40 (3.52, 5.51)	12.92	< 0.001
Continuous (per one-point increase) (2, 10, 25)	3	94.3	< 0.001	0.78 (0.63, 0.97)	−2.23	0.026
Polypharmacy (13, 21, 22)	3	60.6	0.079	4.37 (2.11, 9.06)	3.97	< 0.001
Weakness (3, 21, 24, 29)	4	54.3	0.087	4.28 (2.28, 8.04)	4.52	< 0.001
Malnutrition (10, 13, 22, 26)	4	89.8	< 0.001	1.71 (0.76, 3.84)	1.30	0.194
Cognitive impairment (3, 24, 26)	3	55.3	0.107	1.54 (1.19, 2.00)	3.30	0.001
Oral health (3, 13, 24, 27)	4	73.8	0.010	0.87 (0.84, 0.91)	−6.44	< 0.001

### 3.4 Sensitivity analysis

**Figure 3:**
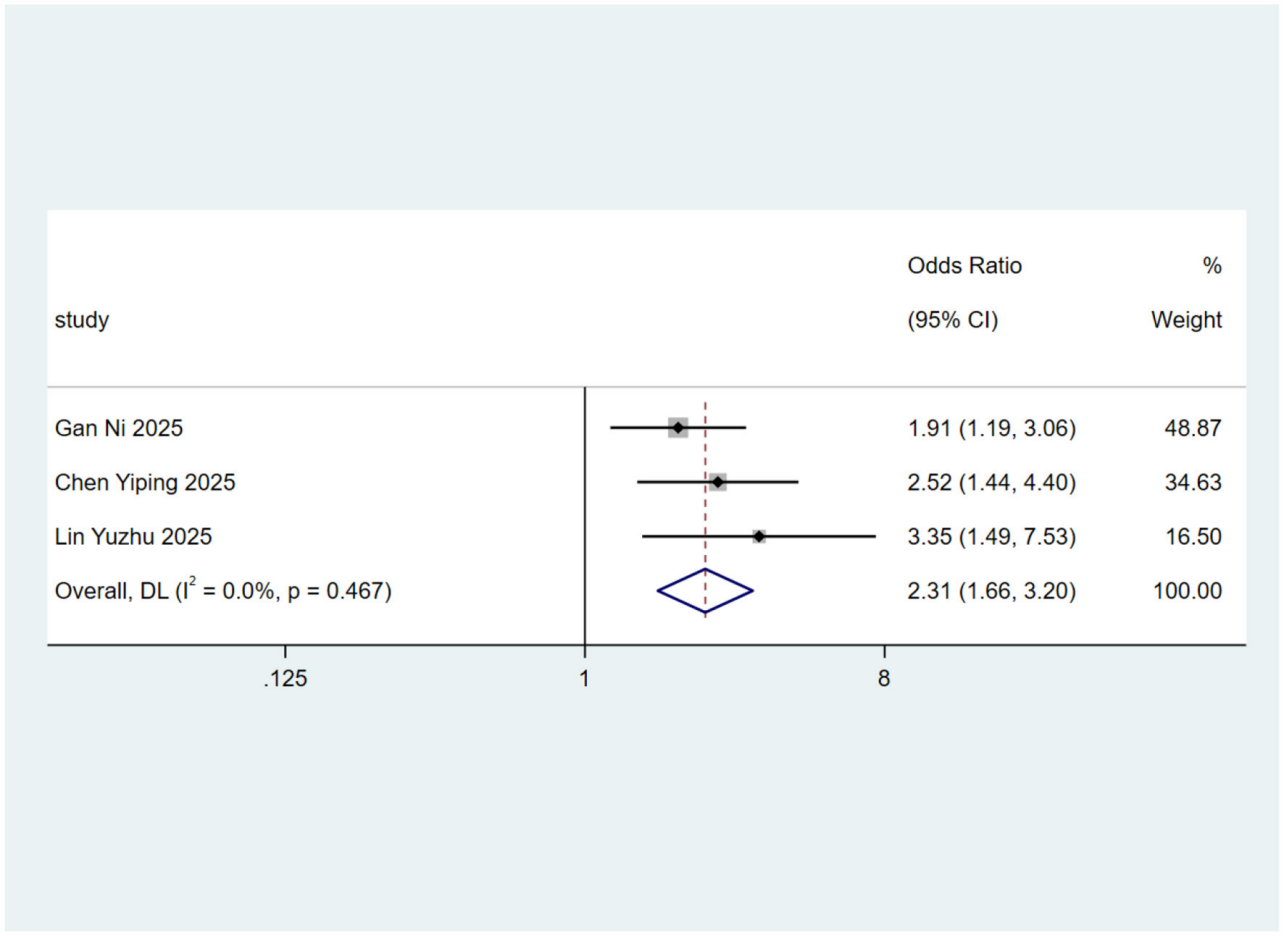
Forest map for sensitivity analysis of malnutrition in predicting dysphagia. Weights are from random-effects model.

Sensitivity analyses were performed for predictors with high heterogeneity by sequentially excluding individual studies. The results indicated that the pooled effect of malnutrition was strongly influenced by a single study: after excluding Liu et al. (10), heterogeneity dropped sharply from *I*^2^ = 89.8%−0%, and the *Q*-test was no longer significant, identifying this study as the primary source of heterogeneity. Using a random-effects model for the remaining studies, malnutrition was confirmed as an independent predictor of dysphagia (OR = 2.31, 95% CI: 1.66–3.20, *p* < 0.001), with Liu et al. (10) representing a key outlier contributing to heterogeneity. Forest plots are presented in Figure 3.

## 4 Discussion

Currently, the identification of dysphagia largely relies on incidental observations by clinical staff, which may delay the initiation of interventions and allow aspiration-related complications to accumulate (30). Implementing validated predictive models for risk stratification at the time of admission could enable precise identification of high-risk individuals before overt swallowing difficulties manifest. This approach would facilitate timely multidisciplinary interventions, including speech-language pathologist (SLP) consultations, dietary texture modifications, optimization of medication formulations, and enteral nutrition planning, potentially shortening the interval between risk exposure and effective intervention (15).

### 4.1 Performance and bias risk analysis of predictive models

This study systematically reviewed 17 investigations, reporting a total of 30 predictive models for dysphagia risk in older adults, all developed between 2021 and 2025, predominantly using data from Chinese populations. The overall performance of these models is critically determined by study design, modeling approaches, and predictive efficacy. The area under the receiver operating characteristic curve (AUC), which intuitively reflects overall discriminative accuracy, has become the most widely used comprehensive metric in the field, with values approaching 1 indicating near-optimal model performance. Pooled analysis of seven studies yielded an AUC of 0.82 (95% CI: 0.77–0.88), suggesting that current algorithms demonstrate good accuracy in predicting dysphagia among older adults. Notably, according to PROBAST assessments, all included studies exhibited significant risk of bias, which may compromise the reliability of the findings.

Among the included studies, the vast majority employed retrospective or cross-sectional designs, with only one prospective study (23). This imbalance in study design exposes potential biases related to sample source, population representativeness, and data acquisition. Retrospective analyses rely on existing records, which are often not collected for research purposes, and are therefore susceptible to missing fields, inconsistent variable definitions, and data entry errors. High rates of missing predictors further exacerbate bias. Critically, the temporal sequence of exposure and outcome is often unclear in retrospective designs, limiting causal inference. In contrast, prospective cohorts systematically collect predictor information before events occur, ensuring complete and standardized variables while enabling real-time outcome tracking, thereby enhancing data quality and model precision.

To strengthen the scientific rigor and causal inference of predictive models, future research should prioritize prospective cohort designs, nested case–control studies, or analyses based on high-quality registry databases, following established international methodological frameworks (18). Nonetheless, prospective studies face practical challenges, including high costs, difficult follow-up, and participant attrition. A staged, multi-level research strategy is therefore recommended: in the initial phase, cross-sectional data can be used to identify potential risk factors and construct preliminary predictive models; subsequently, prospective data can validate model stability and predictive accuracy while clarifying the temporal relationship between exposure and outcome; finally, when resources permit, interventional studies or real-world data can be integrated to optimize and externally validate models, thereby enhancing their clinical applicability and translational value (31).

Regarding predictor variables, researchers should select variables by balancing clinical relevance, accessibility, feasibility, and cost-effectiveness (32). For example, in the study by Peng et al. (3), multiple assessment scales were included (frailty, ADL, oral health, depression, cognition, etc.). While these scales theoretically provide a comprehensive representation of older adults' physiological and functional status, several practical challenges may arise. First, the increased number and intensity of assessments raise both time and labor costs (33), potentially reducing participant compliance and increasing missing data, particularly among cognitively or physically impaired older adults. Second, reliance on objective measurements requiring specialized instruments or training (e.g., muscle mass evaluation, professional oral health scoring) limits applicability in primary care or resource-constrained settings. Third, overlapping information across multiple scales (e.g., frailty, sarcopenia, and ADL in functional domains) may introduce multicollinearity and reduce interpretability, making models complex but less practical. Moreover, none of the studies reported whether blinding was applied during the assessment of predictors or outcomes, which can introduce assessor bias (34). Finally, the temporal interval between predictor measurement and outcome occurrence was often unclear. Collecting predictors after outcome events may inflate associations, increasing the risk of false positives, whereas overly long intervals can attenuate predictor–outcome relationships (34). These methodological limitations may compromise the reliability and validity of predictive model results.

In existing studies, most predictive models exhibit a high risk of bias, attributable to several key factors. First, the handling of continuous variables is frequently suboptimal. Many studies arbitrarily categorized continuous variables (e.g., age, number of missing teeth) without sufficient statistical justification (2, 3, 10, 13, 14, 19–27, 29). This approach not only reduces information content but may also impair model discrimination and calibration. Retaining variables in their continuous form preserves data granularity, and when categorization is necessary, the statistical rationale and cut-off selection criteria should be clearly specified (32, 35). Second, insufficient sample size represents a major limitation. Approximately half of the studies did not meet the recommended events-per-variable (EPV) threshold of ≥20 (13, 19–22, 25, 29). EPV is a critical metric for assessing sample adequacy and preventing model overfitting; an EPV of 10–20 is generally considered minimal, while values above 20 enhance model stability and generalizability (31, 36). Third, transparency in handling missing data is largely inadequate. Most studies failed to report strategies for addressing missing values (2, 10, 13, 14, 19–23, 26–29). Only one study mentioned supplementary data collection but lacked a systematic protocol or detailed results (25). Such methodological omissions compromise both transparency and reproducibility. Fourth, model validation is generally insufficient. Only two studies performed both internal and external validation (10, 15), while the remaining fourteen conducted internal validation alone (2, 3, 13, 14, 19, 20, 22–29). The absence of external validation raises concerns about generalizability and reliability (32, 37). External validation is crucial for assessing model performance across diverse populations, healthcare settings, and data sources; without it, models may perform substantially worse in real-world clinical contexts, limiting their translational value (32). Fifth, reporting of model performance remains incomplete. Only nine studies provided at least one measure of sensitivity, specificity, or overall accuracy (2, 3, 14, 20, 22, 24, 27–29), and most results were not comprehensively presented. Inadequate reporting impedes quality assessment, cross-study comparison, and interpretability, thereby reducing the reliability of applying these models in clinical decision-making (32). Finally, all studies assumed independence among participants, failing to adjust for clustering effects across centers or institutions, and did not account for competing risks such as death, loss to follow-up, or transfers. These methodological oversights may bias estimates and artificially inflate model performance (18).

In the outcome domain, the assessment of predictors and outcomes may be susceptible to information contamination or data leakage, which can artificially inflate model performance (32). Most studies evaluated swallowing function using standardized instruments such as the WST, SSA, or FOIS, with clearly defined cut-off points. However, studies generally did not report whether outcome assessors were blinded to predictor information, nor did they specify the temporal sequence between predictor measurement and outcome assessment, thereby increasing the risk of outcome-related bias.

### 4.2 Analysis of common predictors in risk prediction models

Meta-analyses including three or more studies indicate that advanced age, smoking history, impaired functional independence, polypharmacy, frailty, malnutrition, cognitive impairment, and poor oral health are independent risk factors for dysphagia.

Several studies have highlighted the close association between age-related physiological degeneration and diminished swallowing function (2, 3, 13, 19, 20, 23, 24, 27, 29). With advancing age, the mass and elasticity of the hyoid bone and swallowing-related musculature decline, while muscle strength and contraction velocity decrease, directly impairing bolus propulsion (2, 4). Concurrent degeneration of central and peripheral neural structures, along with reduced motor unit numbers and slowed conduction velocity, compromises the temporal coordination of swallowing and airway protective reflexes (10). Additional age-related changes—including reduced salivary secretion, tooth loss, elevated oropharyngeal sensory thresholds, and diminished olfactory and gustatory sensitivity—further hinder bolus formation and swallowing initiation. Cervical spondylosis and prolonged forward head posture can mechanically compress the upper esophageal sphincter, increasing resistance during bolus passage (2). Moreover, older adults may have limited awareness of swallowing dysfunction, leading to underreporting or delayed medical consultation, which postpones early diagnosis and intervention (10).

Three studies identified smoking history as a predictor of dysphagia risk in older adults (19, 22, 27). The myriad toxic compounds released through smoking chronically irritate the pharyngeal and esophageal epithelium, damaging mucosal barrier structures and triggering persistent inflammation. This leads to diminished local sensation and weakened protective reflexes, compromising both the safety and efficiency of swallowing (19, 22). Long-term smoking is also closely linked to poor oral health, including increased risk of gingivitis, periodontitis, and tooth loss, which further impairs masticatory function (27). Moreover, smoking promotes the development and progression of chronic metabolic conditions such as hypertension and diabetes, heightening systemic inflammatory burden. The presence of multiple comorbidities is a significant risk factor for dysphagia in older adults (27).

Meanwhile, impaired functional independence is recognized as a significant risk factor for dysphagia (2, 3, 10, 21, 23–25). Among older adults with severe chronic illnesses or functional dependence, oral health is often neglected (38). Studies indicate that reduced self-care capacity and increased reliance on assistance for activities of daily living are independently and significantly associated with the occurrence of dysphagia (39). Although the motor control mechanisms of the oropharyngeal system differ from those of the limbs, diminished daily functional abilities may indirectly compromise oral function, thereby elevating the risk of swallowing difficulties (39).

Regarding polypharmacy (13, 21, 22), the study by Xie et al. (21) reported that older adults taking three or more medications had approximately a 4.5-fold increased risk of dysphagia. Potential mechanisms include: (1) cumulative adverse effects from multiple drugs, which may suppress appetite and trigger abnormal eating behaviors; (2) sedatives and antidepressants that can directly inhibit the swallowing reflex; and (3) anticholinergic medications that reduce salivary secretion, leading to dry mouth and impaired mastication (13, 21, 22). However, existing studies only considered the total number of medications as the exposure variable, without stratifying by drug class. Given that different pharmacological types may exert heterogeneous effects on swallowing function, this represents a notable limitation of current predictive models.

Frailty and malnutrition are key predictors of dysphagia (3, 10, 13, 21, 22, 24, 26, 29). Unlike the mild decline in swallowing function that may occur with normal aging, frailty compromises both the efficiency and safety of swallowing. Clinical studies (40) have shown that among hospitalized patients aged 50 and older, frail individuals exhibit a significantly higher incidence of dysphagia compared with non-frail counterparts. Dysphagic patients also experience longer hospital stays, higher medical costs, increased rates of unplanned discharge, and a greater incidence of medical complications. Impaired swallowing can lead to inadequate nutritional intake, and malnutrition further diminishes the strength and coordination of swallowing muscles. Deficiencies in vitamins such as B12 and D exacerbate neuromuscular conduction impairments, accelerating the decline in swallowing function (13). This, in turn, promotes the onset and progression of frailty (3, 21, 24, 29, 40), creating a vicious cycle.

Cognitive impairment is another significant risk factor for dysphagia (3, 24, 26). Declines in cognitive function can lead to progressive deterioration of motor abilities and muscle function, thereby increasing the likelihood of swallowing difficulties. Cognitive deficits also compromise patients' control and judgment during swallowing, heightening the risk of unsafe swallowing behaviors such as aspiration (3, 24, 26). Additionally, impaired cognition can disrupt the coordination of oropharyngeal musculature, hindering the smooth execution of swallowing. Some patients may also exhibit reduced food intake or refusal to eat due to cognitive decline, which can exacerbate malnutrition and further impair swallowing function (26).

Finally, common oral health problems such as tooth loss and dental caries can disrupt normal mastication and food processing, reducing the efficiency of bolus formation and transport. Additionally, imbalances in the oral microbiome may trigger localized inflammatory responses, impairing the functional status and neuromuscular coordination of oral and pharyngeal tissues, thereby interfering with the swallowing process (13).

### 4.3 Implications for future research

Future research should focus on enhancing the methodological rigor and clinical applicability of prediction models for dysphagia in older adults. Priority should be given to prospective cohort designs, adequate sample sizes, and transparent handling of missing data to minimize bias. In addition, external validation across diverse populations and healthcare settings is essential for evaluating model generalizability and ensuring reliable clinical implementation. Predictor selection should emphasize clinical feasibility and avoid excessive reliance on multiple complex assessment scales that may limit real-world applicability. More detailed characterization of medication classes, functional status, and nutritional indicators may further improve model performance. Where feasible, the use of standardized outcome measures and validated assessment tools would enhance comparability across studies and strengthen the robustness of predictive models. Importantly, neurological disorders warrant greater attention in future investigations. Swallowing is a complex sensorimotor process regulated by multiple brain regions, with the underlying neural control network involving the cerebral cortex, subcortical structures, brainstem, and cerebellum (41). The primary sensorimotor cortex, insula, and anterior cingulate cortex may participate in the initiation of swallowing and the coordination of oral and pharyngeal phase movements, interacting with brainstem swallowing control centers through descending neural pathways (42). The medullary swallowing central pattern generator integrates sensory inputs from the cortex and cranial nerves to generate and modulate fundamental motor patterns of swallowing musculature, although its function remains subject to multilevel feedback regulation within the central nervous system (42). Neurological injury may disrupt the normal function of the swallowing neural network, leading to delayed reflex initiation, impaired motor coordination, and weakened airway protective mechanisms, thereby increasing the risk of dysphagia and aspiration (41, 42). Therefore, future studies may further explore incorporating clinical features related to neurological diseases into prediction models, such as disease type, severity, and functional status, to improve the accuracy of dysphagia risk assessment in older adults.

### 4.4 Limitations

This review has several limitations that should be considered when interpreting the findings. First, among the 17 included studies, 15 were conducted in China. The concentration of studies from a single country may limit the external generalizability of the results, particularly given variations in healthcare systems, resource allocation, patient characteristics, and healthcare-seeking behaviors across regions. Second, despite systematically searching multiple major databases, some relevant studies may have been missed, particularly those published in regional journals or gray literature, which may result in incomplete coverage of the available evidence. In addition, only studies published in English and Chinese were included, introducing potential language bias. Third, the definition and assessment of dysphagia varied across the included studies. Some studies identified dysphagia based on self-reported symptoms, whereas others relied on clinical swallowing evaluations. Since large-scale epidemiological investigations and early prediction modeling studies in older populations rarely employ instrumental diagnostic examinations such as VFSS, the inclusion strategy of this review reflects current evidence derived from real-world screening and clinical assessment practices. Fourth, the included studies involved diverse clinical settings, ranging from community-dwelling older adults to hospitalized patients and disease-specific populations. The predictive performance of risk factors and models may therefore vary across clinical contexts, potentially limiting the comparability and universality of the pooled results. Finally, although neurological disease is considered an important risk factor for dysphagia, subgroup meta-analysis stratified by neurological disease status was not conducted due to the limited number of eligible studies reporting disease-specific data. Accordingly, this review primarily reflects common predictors of dysphagia in heterogeneous older populations rather than disease-specific predictive effects. These limitations should be considered when interpreting the clinical implications of the findings.

## 5 Conclusion

The results of this review indicate that the evaluated predictive models for dysphagia in older adults generally demonstrate good predictive performance and support clinicians in the timely identification of high-risk individuals. However, the models collectively exhibit a high risk of bias, which underscores the need to integrate clinical judgment and professional expertise when applying them in practice. Future model development should adhere to PROBAST guidelines and incorporate real-world clinical contexts to optimize study design and methodological quality. Only through systematic and rigorous external validation can predictive tools be established that are clinically practical, scientifically robust, and operationally valuable.

## Data Availability

The original contributions presented in the study are included in the article/Supplementary material, further inquiries can be directed to the corresponding author.
