# Supplementary material for: Predictive models for dysphagia in older adults: a systematic review and meta-analysis
Source: Front Public Health. 2026 Apr 2;14:1792380. doi: 10.3389/fpubh.2026.1792380 (PMC13082982; doi:10.3389/fpubh.2026.1792380)

**Predictive Models for Dysphagia in Older Adults: a Systematic Review and Meta-Analysis**

**Table 1** Search strategy

| Database | Search strategies |
| --- | --- |
| PubMed | #1: "Aged"[Mesh] OR aged[tiab] OR elderly[tiab] OR "older adult*"[tiab] OR "older people"[tiab] OR "older population"[tiab] OR geriatric[tiab]  #2: dysphagia[Mesh] OR dysphagia[tiab] OR "swallowing difficulty"[tiab] OR "swallowing disorder"[tiab] OR "swallowing dysfunction"[tiab] OR "deglutition disorder"[tiab] OR "deglutition disorders"[tiab]  #3: "Models, Statistical"[Mesh] OR "Machine Learning"[Mesh] OR "Risk Assessment"[Mesh] OR predict*[tiab] OR "prediction model*"[tiab] OR "risk score"[tiab] OR "risk assessment"[tiab] OR "risk stratification"[tiab] OR nomogram*[tiab] OR "scoring system"[tiab] OR "machine learning"[tiab] OR "decision tree*"[tiab] OR "random forest"[tiab] OR "logistic regression"[tiab] OR "neural network*"[tiab] OR "deep learning"[tiab] OR XGBoost[tiab] OR "support vector"[tiab] OR algorithm*[tiab]  #4: #1 AND #2 AND #3 |
| Web of science | #1: TS=(aged OR elderly OR "older adult*" OR "older people" OR "older population" OR geriatric)  #2: TS=(dysphagia OR dysphagia OR "swallowing difficulty" OR "swallowing disorder" OR "swallowing dysfunction" OR "deglutition disorder" OR "deglutition disorders")  #3: TS=(predict* OR "prediction model*" OR "risk score*" OR "risk assessment" OR "risk stratification" OR nomogram* OR "scoring system*" OR "machine learning" OR "decision tree*" OR "random forest" OR "logistic regression" OR "neural network*" OR "deep learning" OR XGBoost OR "support vector" OR algorithm*)  #4: #1 AND #2 AND #3 |
| Cochrane Library | #1: (aged or elderly or older adult* or older people or older population or geriatric):ti,ab,kw  #2: (dysphagia or swallowing difficulty or swallowing disorder* or swallowing dysfunction or deglutition disorder*):ti,ab,kw  #3: (predict OR prediction OR "prediction model" OR "risk score" OR "risk assessment" OR "risk stratification" OR nomogram OR "scoring system" OR "machine learning" OR "decision tree" OR "random forest" OR "logistic regression" OR "neural network" OR "deep learning" OR XGBoost OR "support vector" OR algorithm):ti,ab,kw  #4: #1 AND #2 AND #3 |
| CNKI | #1: TKA=老年 + 高龄 + 老龄 + 老年人 + 老人  #2: TKA=吞咽困难 + 吞咽障碍 + 吞咽功能障碍  #3: TKA=预测 + 预测模型 + 预测工具 + 筛查工具 + 风险预测 + 风险评估 + 风险评分 + 列线图 + 机器学习  #4: #1 AND #2 AND #3 |
| WanFang | #1: 题名或关键词=老年 OR 高龄 OR 老龄 OR 老年人 OR 老人  #2: 题名或关键词=吞咽困难 OR 吞咽障碍 OR 吞咽功能障碍  #3: 题名或关键词=预测 OR 预测模型 OR 预测工具 OR 筛查工具 OR 风险预测 OR 风险评估 OR 风险评分 OR 列线图 OR 机器学习  #4: #1 AND #2 AND #3 |
| Sinomed | #1老年 [摘要] OR 高龄 [摘要] OR 老龄 [摘要] OR 老年人 [摘要] OR 老人[摘要]  #2吞咽困难 [摘要] OR 吞咽障碍 [摘要] OR 吞咽功能障碍 [摘要]  #3预测 [摘要] OR 预测模型 [摘要] OR 预测工具 [摘要] OR 筛查工具 [摘要] OR 风险预测 [摘要] OR 风险评估 [摘要] OR 风险评分 [摘要] OR 列线图 [摘要] OR 机器学习 [摘要]  #4: #1 AND #2 AND #3 |
| VIP | #1: 题名或关键词=老年 OR 高龄 OR 老龄 OR 老年人 OR 老人  #2: 题名或关键词=吞咽困难 OR 吞咽障碍 OR 吞咽功能障碍  #3: 题名或关键词=预测 OR 预测模型 OR 预测工具 OR 筛查工具 OR 风险预测 OR 风险评估 OR 风险评分 OR 列线图 OR 机器学习  #4: #1 AND #2 AND #3 |

**Figure 1** Forest plot of age


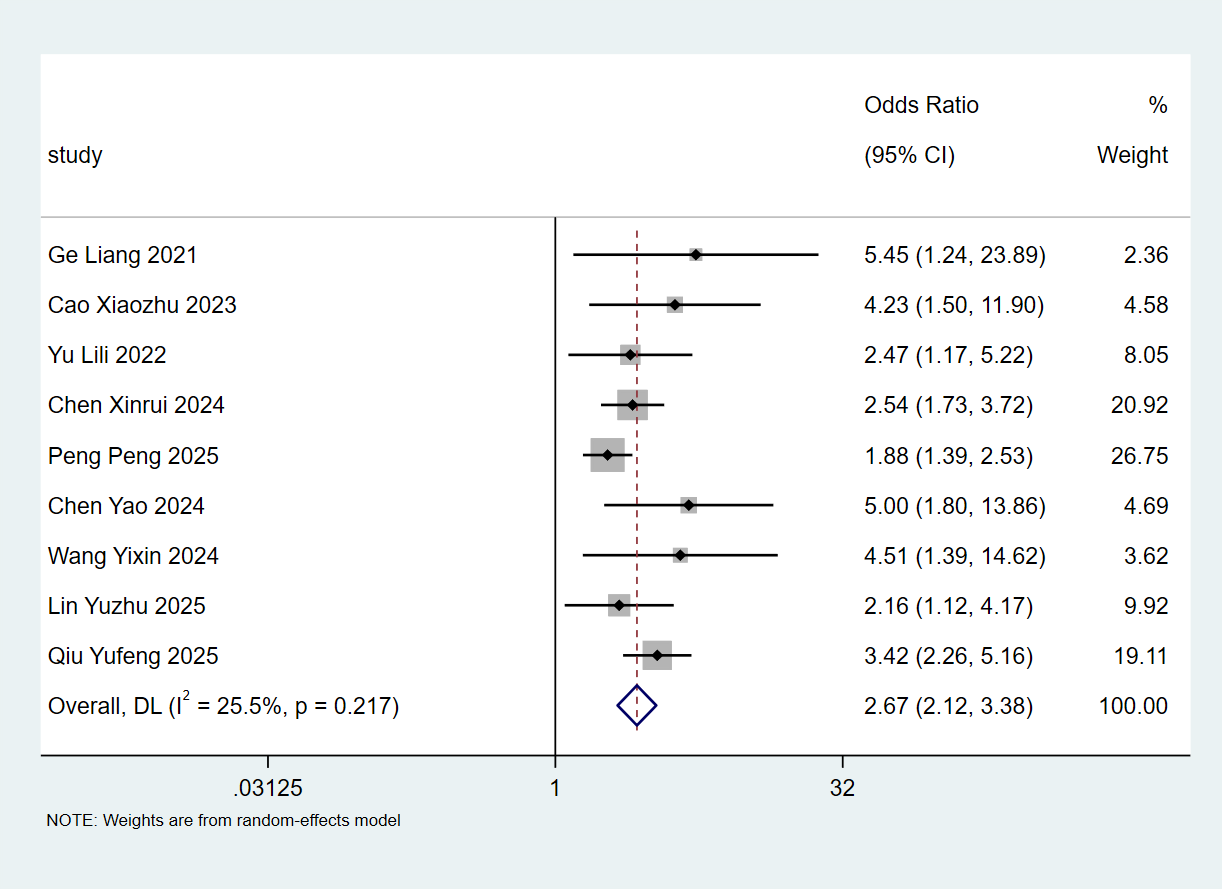


**Figure 2** Forest plot of smoking history


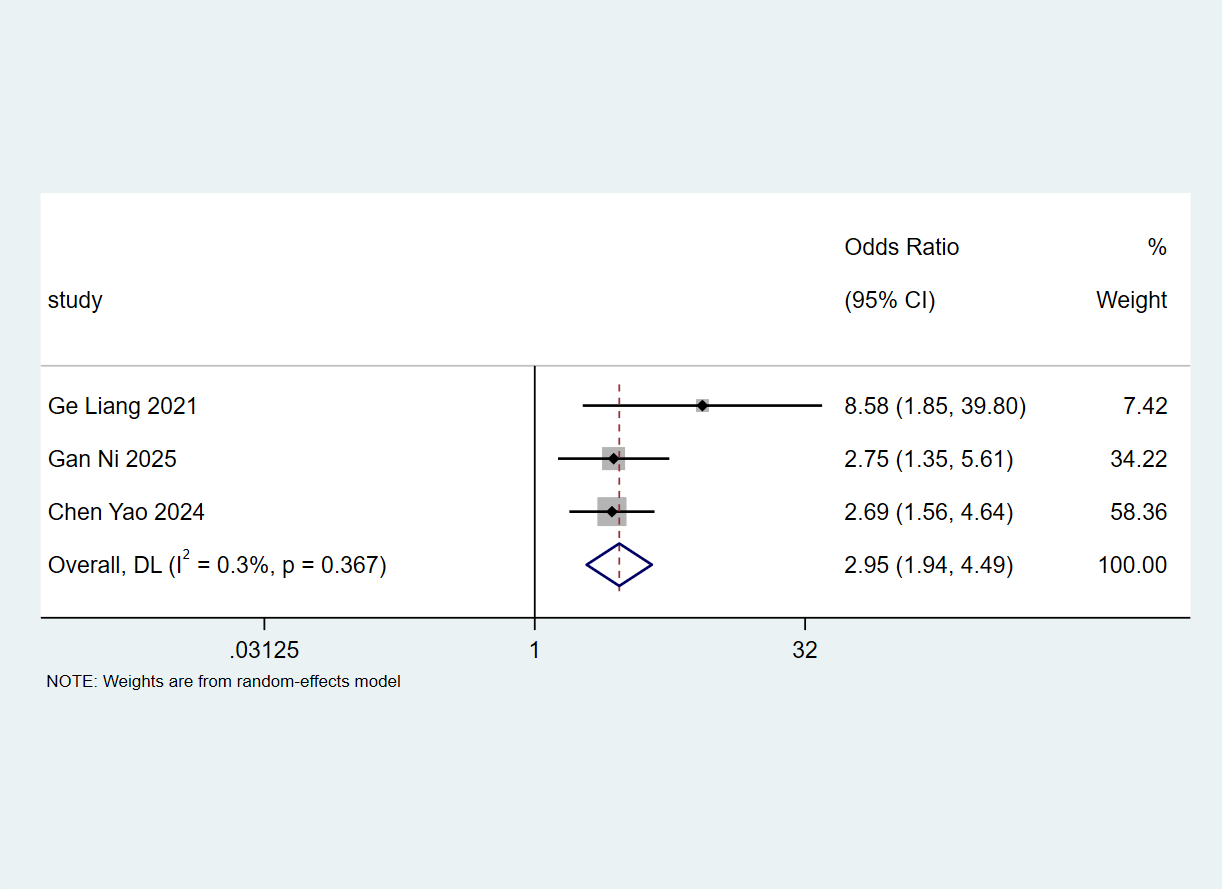


**Figure 3** Forest plot of self-care ability (categorical variable)


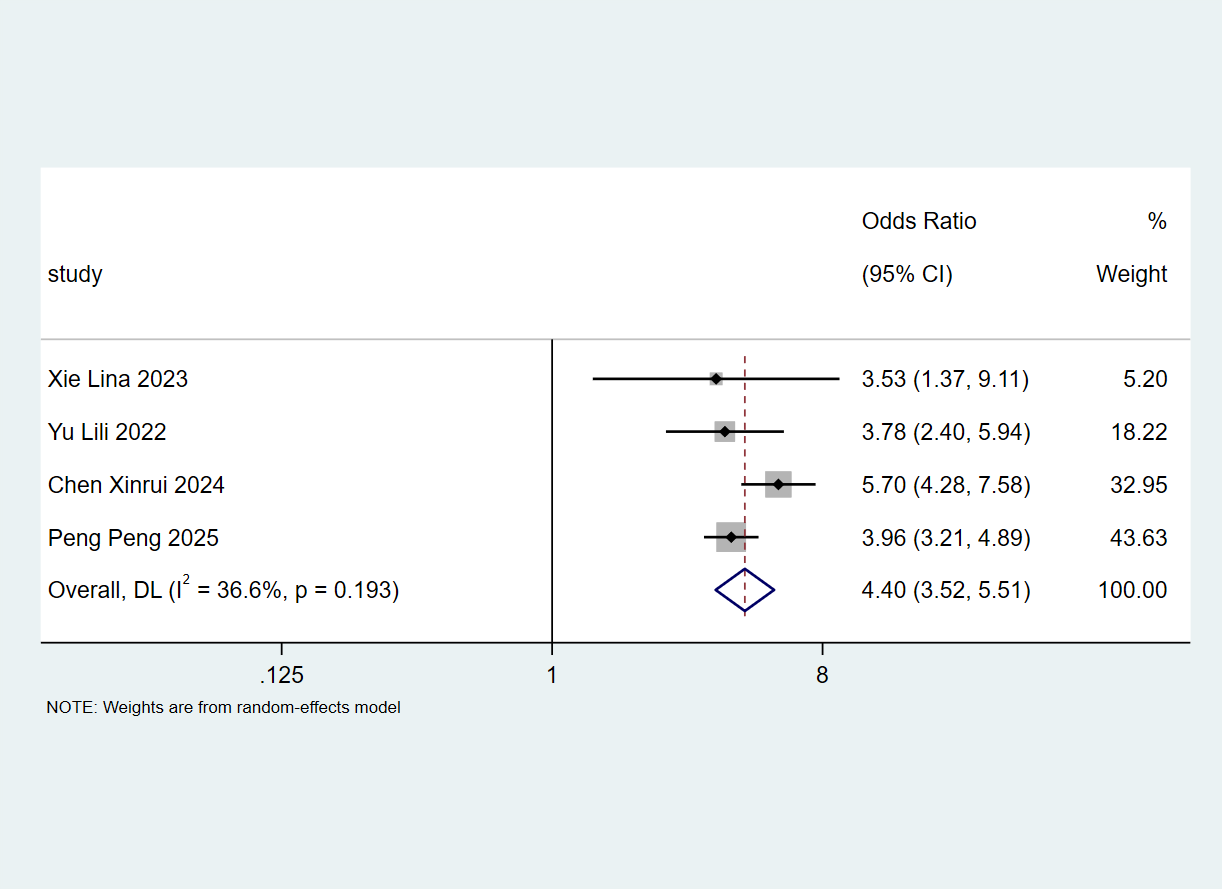


**Figure 4** Forest plot of self-care ability (continuous variable)


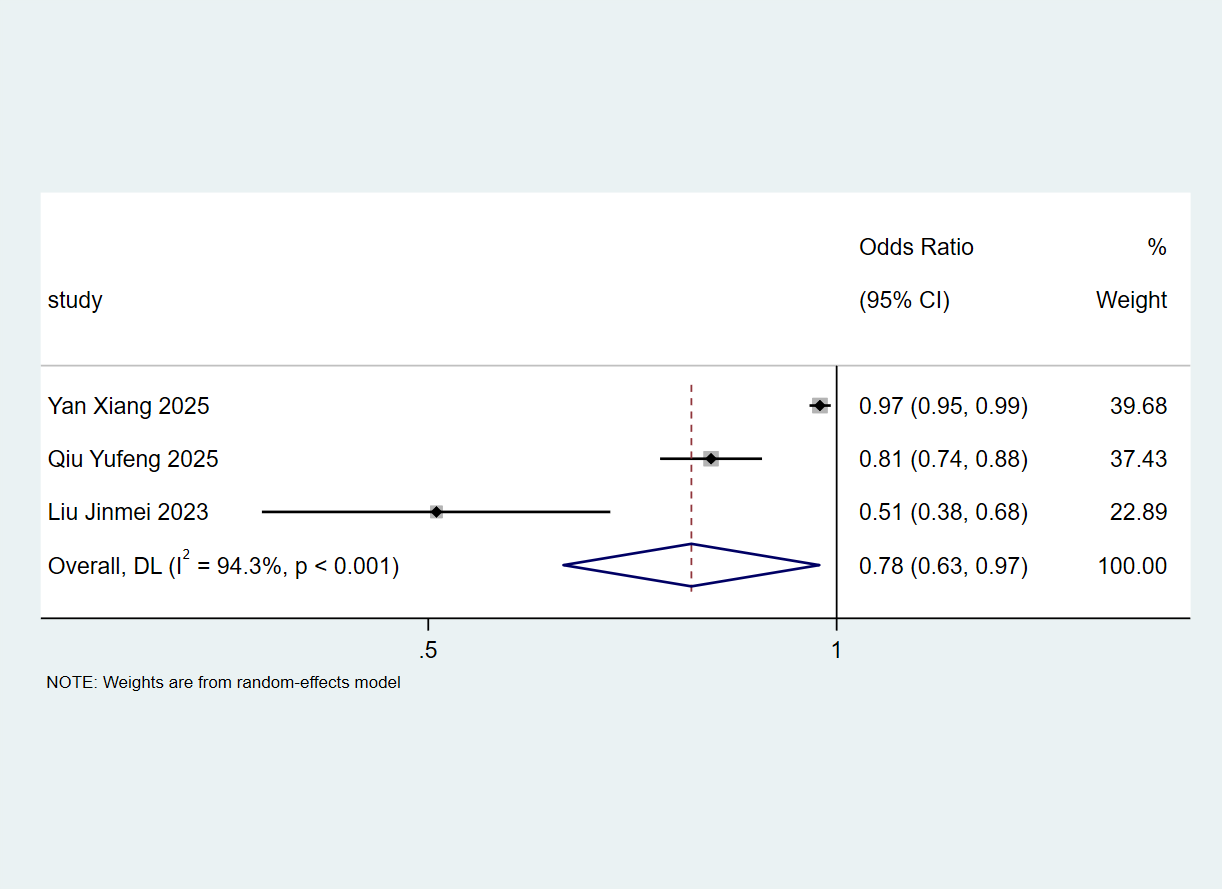


**Figure 5** Forest plot of polypharmacy


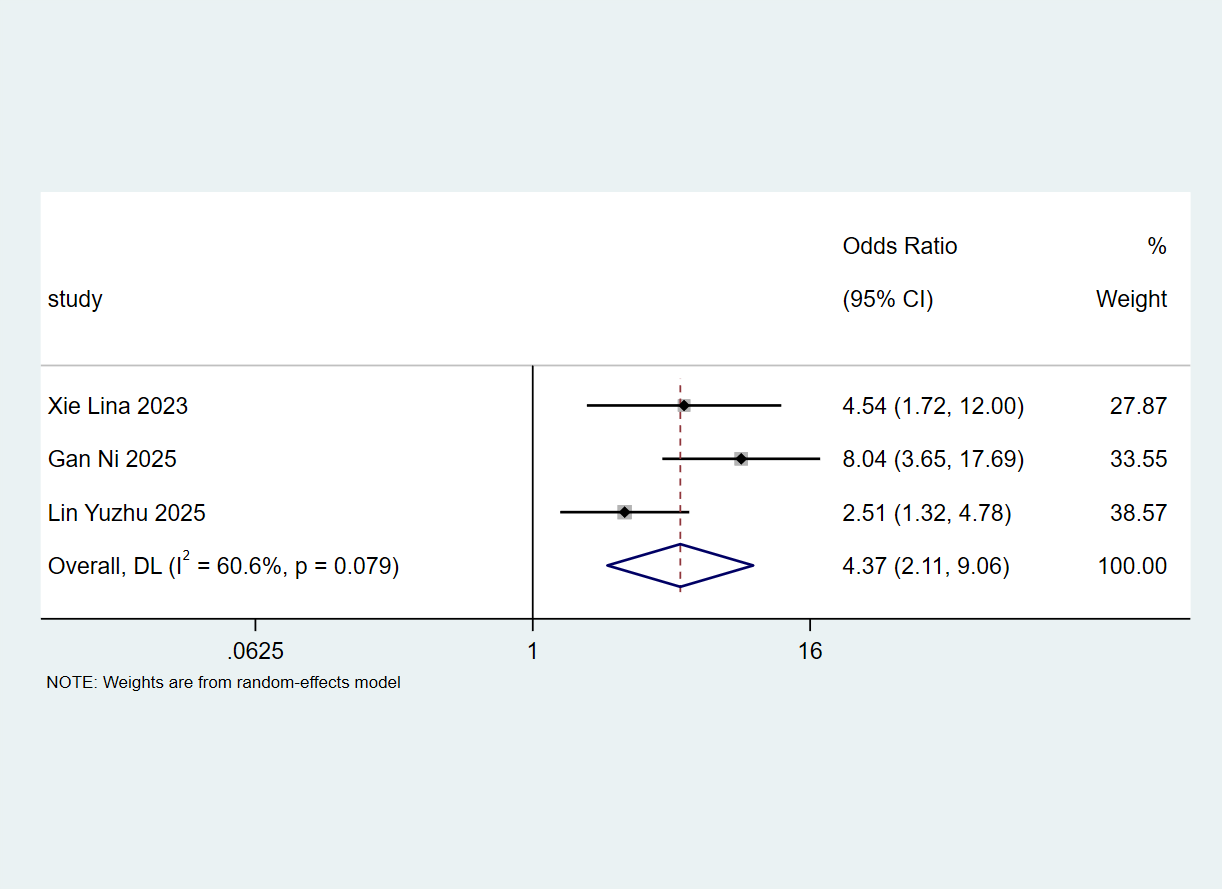


**Figure 6** Forest plot of weakness


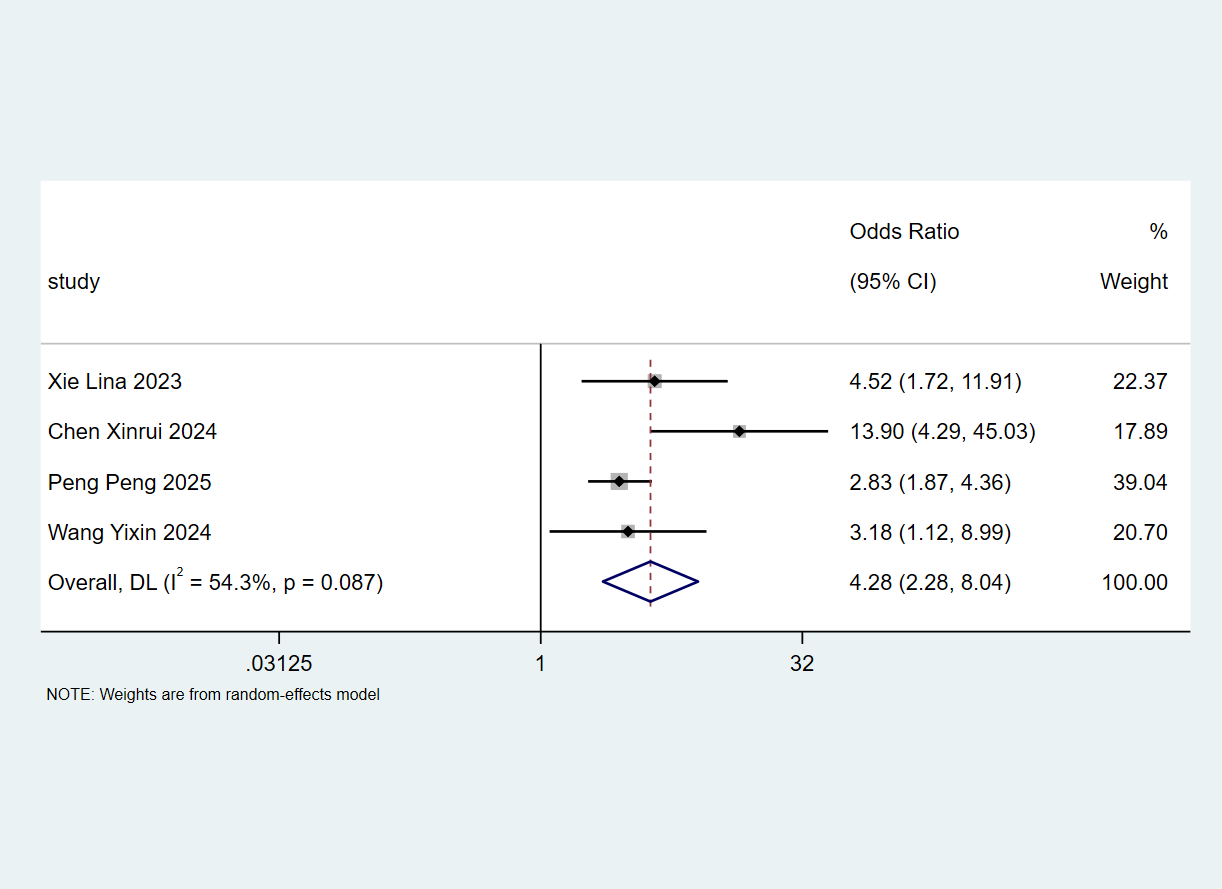


**Figure 7** Forest plot of malnutrition


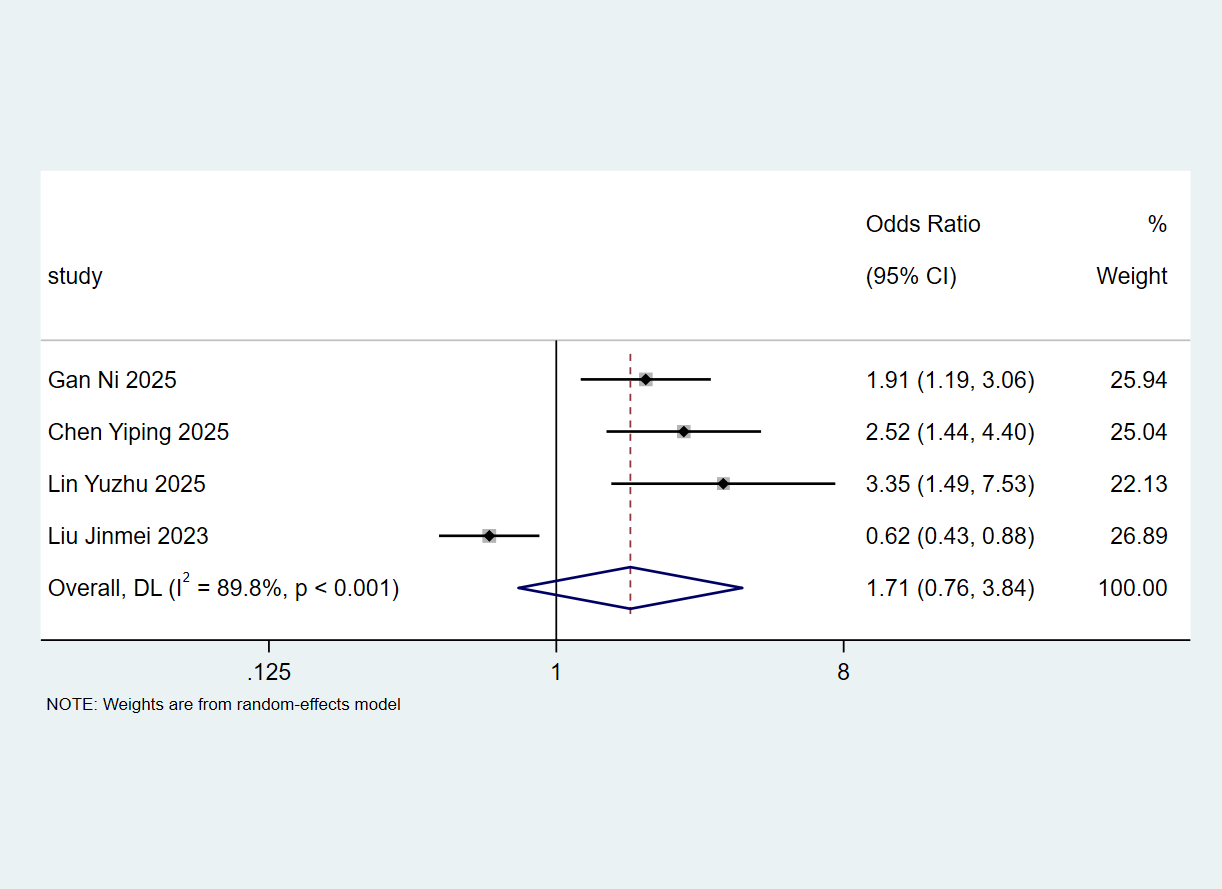


**Figure 8** Forest plot of cognitive impairment


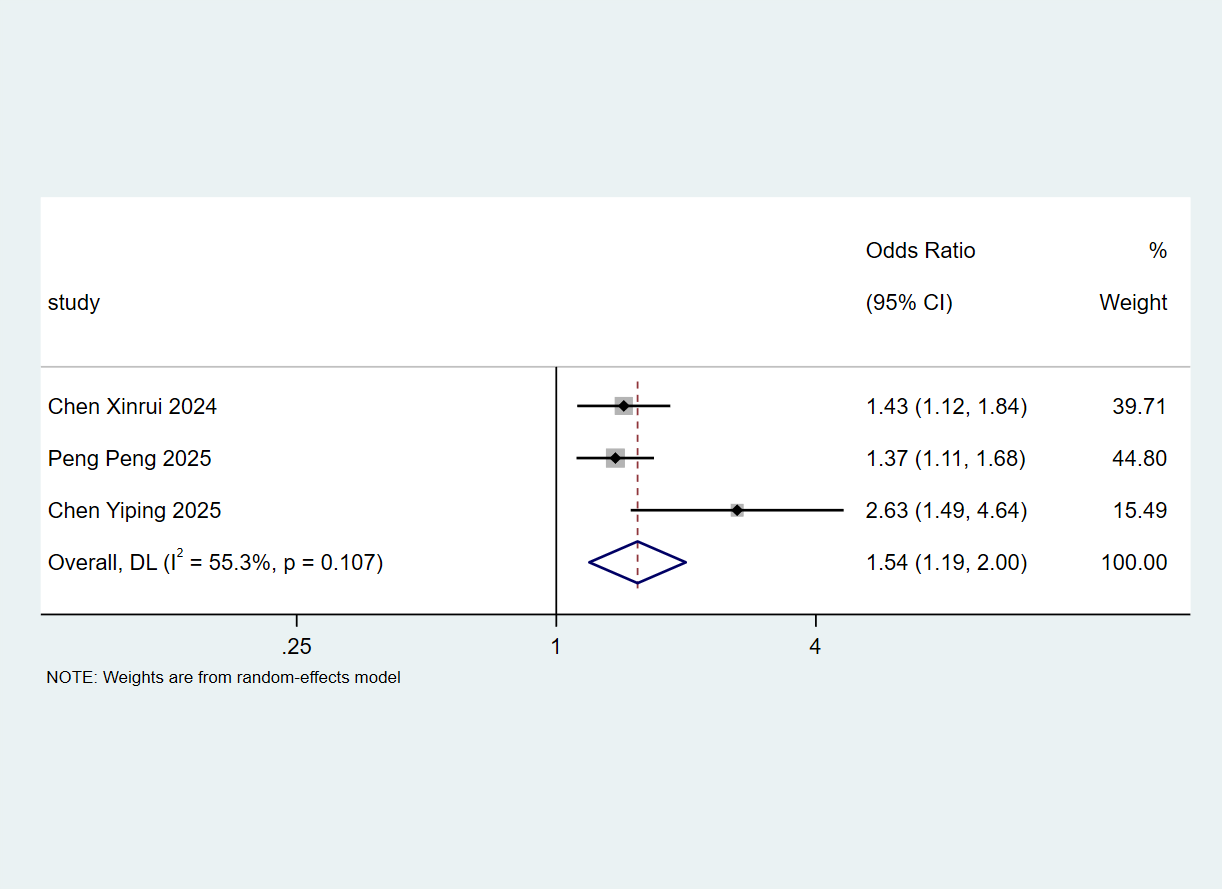


**Figure 9** Forest plot of oral health


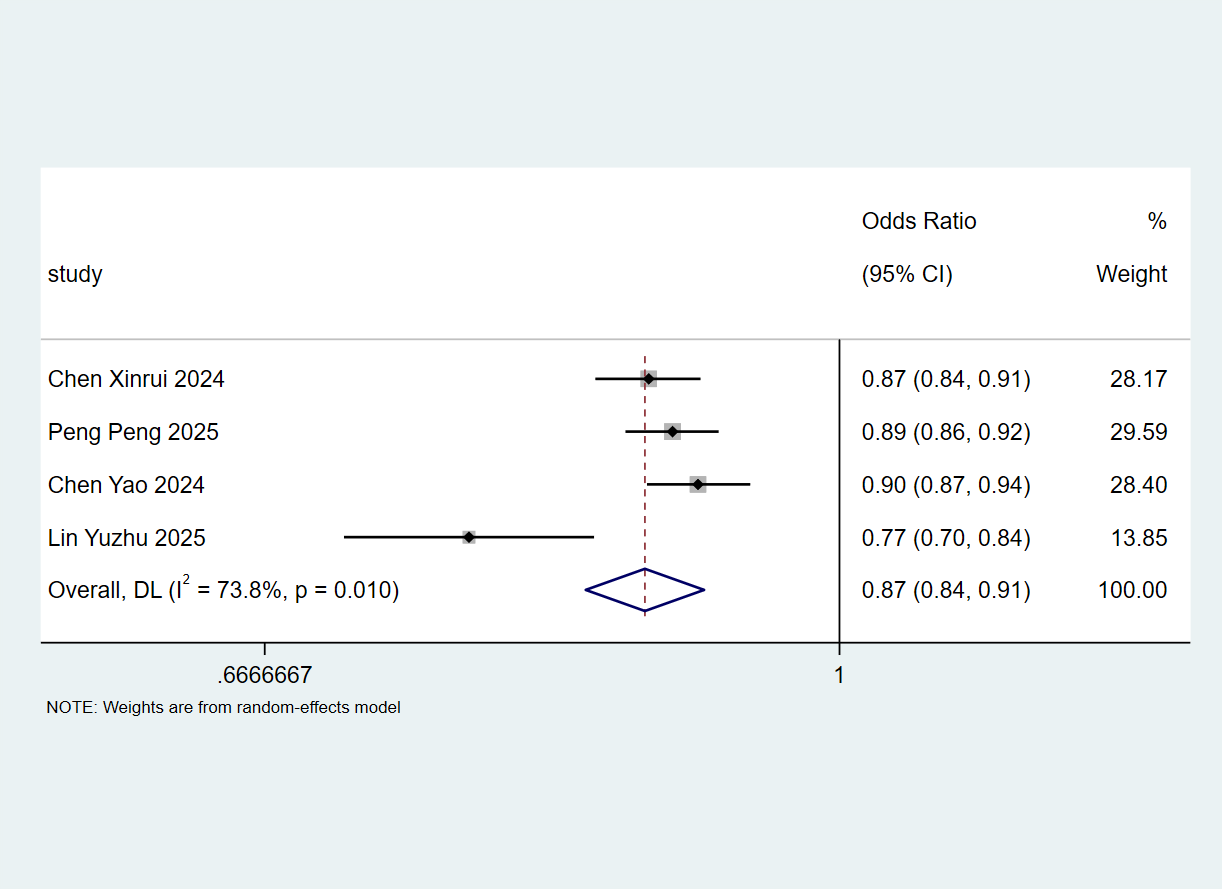

Supplement: Supplementary file 1 [file Supplementary_file_1.docx]
